# Electrochemical and Bioelectrochemical Sensing Platforms for Diagnostics of COVID-19

**DOI:** 10.3390/bios13030336

**Published:** 2023-03-03

**Authors:** Milena do Prado Ferreira, Sueli Fumie Yamada-Ogatta, César Ricardo Teixeira Tarley

**Affiliations:** 1 Department of Chemistry, State University of Londrina (UEL), Londrina 86051-990, Brazil; 2 Department of Microbiology, State University of Londrina (UEL), Londrina 86051-990, Brazil; 3 National Institute of Science and Technology in Bioanalysis (INCTBio), Institute of Chemistry, State University of Campinas (UNICAMP), Campinas 13083-970, Brazil

**Keywords:** electrochemical methods, SARS-CoV-2, COVID-19 diagnostic, coronaviruses, biorecognition elements, biosensors

## Abstract

Rapid transmission and high mortality rates caused by the SARS-CoV-2 virus showed that the best way to fight against the pandemic was through rapid, accurate diagnosis in parallel with vaccination. In this context, several research groups around the world have endeavored to develop new diagnostic methods due to the disadvantages of the gold standard method, reverse transcriptase polymerase chain reaction (RT-PCR), in terms of cost and time consumption. Electrochemical and bioelectrochemical platforms have been important tools for overcoming the limitations of conventional diagnostic platforms, including accuracy, accessibility, portability, and response time. In this review, we report on several electrochemical sensors and biosensors developed for SARS-CoV-2 detection, presenting the concepts, fabrication, advantages, and disadvantages of the different approaches. The focus is devoted to highlighting the recent progress of electrochemical devices developed as next-generation field-deployable analytical tools as well as guiding future researchers in the manufacture of devices for disease diagnosis.

## 1. Introduction

The world has experienced one of the worst pandemics, caused by COVID-19. The officialization of COVID-19 as a pandemic by the WHO (World Health Organization) took place in March 2020, and to date, 651,918,402 cases and 6,656,601 deaths have been confirmed worldwide. After numerous studies, the Chinese confirmed the emergence of a new coronavirus called severe acute respiratory syndrome coronavirus 2 (SARS-CoV-2 or 2019-nCoV), which presented a very high level of contagion with hospitalization conditions that led patients, most of the time, to death. From this, the worldwide scientific and medical communities mobilized to develop vaccines, treatments, and tests to diagnose the disease [1].

To date, the reported numbers of confirmed cases and deaths in Brazil are 36,226,287 and 693,199, respectively (28 December 2022). According to the numbers reported and due to the daily appearance of new cases, early, quick, and accessible diagnosis, together with vaccination, still proves to be the best way to minimize deaths from this disease [2].

Reverse transcription polymerase chain reaction (RT-PCR) is the main diagnostic modality for COVID-19 (gold standard), with a limit of detection of ~100 copies mL^−1^ and a false-negative rate that can vary up to 37% [3,4,5]. It is a molecular technique that identifies viral RNA through the reverse transcription and amplification of nucleic acid, i.e., a direct quantification of viral RNA [5,6,7]. Unfortunately, due to the long turnaround time, skilled technicians, and expensive equipment, RT-PCR has limitations for screening purposes when a rapid and low-cost diagnosis is expected [8,9].

In this context, alternative tests that are already well established for infectious disease diagnostics, such as ELISA (enzyme-linked immunosorbent assay), LFT (lateral flow test), and LAMP (loop-mediated isothermal amplification), can be good tools in the control and diagnosis of COVID-19. Serological tests are based on the immune response of patients. Therefore, these tests detect antibodies formed after infection [10]. Serological tests are performed mainly by enzyme immunoassay (ELISA), which has a shorter analysis time (1–5 h) and a lower cost. However, the use of these tests would not be the best option for the initial diagnosis of the disease [10,11] since the detectable level of immunoglobulin class A (IgA) and class M (IgM) is approximately reached by the fifth day after the onset of symptoms, followed by an increase in IgG levels [8,12,13]. Additionally, likewise, for PCR tests, qualified personnel are needed [10,11]. Therefore, serological assays commonly aid in the detection of antibodies produced by individuals after viral exposure [14].

The LFT is an immunochromatographic assay that uses immobilized antibodies to detect the antigen collected from samples of nasal and oral swabs, providing answers in a short time (15–30 min) [9]. LAMP is a newer technique that uses nucleic acid amplification at one temperature. Its main advantages compared to RT-PCR are simplicity, speed, lower cost, and the lack of the need to use a thermal cycler, which is essential for conventional PCR [15,16].

In general, it is possible to observe that the main drawbacks of the methods commonly used in the diagnosis of COVID-19 are attributed to their high response time, the need for qualified professionals, as well as the cost per analysis. In view of these inherent challenges, the use of electrochemical diagnostics, using biosensors and chemically modified electrodes, is opportune to overcome these inconveniences, taking into account its advantages, such as operational simplicity, detection, cost-effectiveness, smaller amount of sample required (µL to nL), faster response times (several seconds to minutes), and miniaturization capability. Furthermore, similar to the gold standard technique, electrochemical techniques can offer high sensitivity and selectivity and demonstrate their great potential in the detection of different viruses and their antibodies [17].

Therefore, this review presents advances in electrochemical methods for diagnosing and combating the current viral pandemic, with the aim of contributing to the knowledge and assistance of health professionals as well as motivating researchers to develop new and more efficient methods, mainly for point-of-care diagnostics. Thus, a comprehensive review of the state of the art on the electrochemical sensors and biosensors for the diagnosis of COVID-19 is herein presented. The main aim was to elucidate the concept and operation of each method presented, always seeking to expose the main advantages and disadvantages of the different proposals. For final remarks, current challenges and future perspectives are also described.

## 2. Structure of SARS-CoV-2 in Diagnostic Development and Sampling Methods

**Figure 1:**
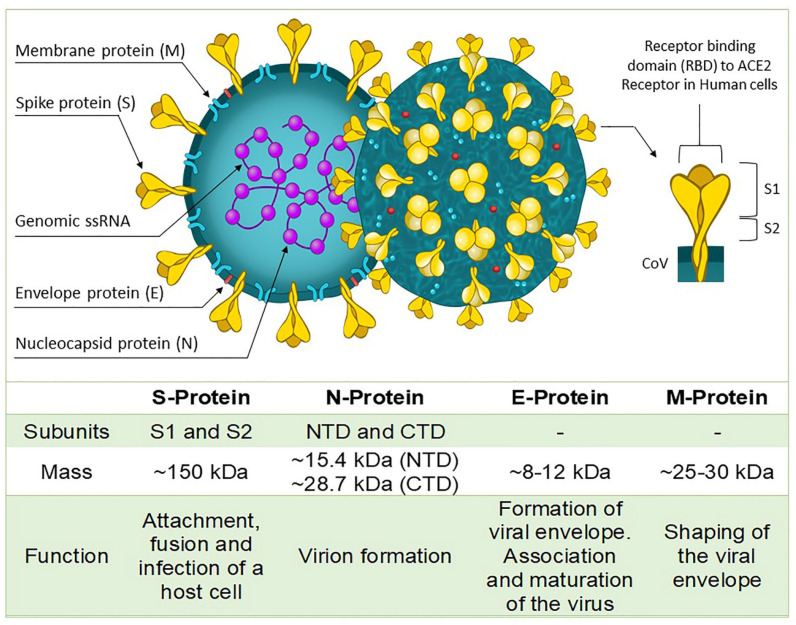
Typical structure of SARS-CoV-2 viral particle.

The response of an electrochemical sensor and a biosensor toward a target analyte intimately depends on the interaction of the sensing platform. Therefore, the design of these devices is based on some structural part of the antigen of interest. The size of SARS-CoV-2 (Figure 1) can range from 50 to 200 nm in diameter. The main components that form its structure are a single strand of ribonucleic acid (RNA) and four proteins, namely the spike protein (s), nucleocapsid protein (N), envelope protein (E), and membrane protein (M) [18].

Most immunoassays detect the presence of antibodies against proteins S or N, which are the main immunogens of SARS-CoV-2. However, electrochemical biosensors developed for SARS-CoV-2, in general, use bioreceptors to detect the presence of proteins S and N. The S-protein has a very important role; it is responsible for binding the virus to the host cell, while the N-protein has as its main function the replication and transcription of the viral genome [19,20].

Thus far, nasopharyngeal swabs are the most used sampling methods for testing for the presence of SARS-CoV-2. This is justified mainly due to the high viral load in the upper respiratory tract. However, studies show that oropharyngeal swab samples show more elevated and stabilized levels of SARS-CoV-2 RNA than nasal samples during the entire infection [21].

Other matrices have been evaluated for detecting SARS-CoV-2, such as urine, blood, stool, and saliva, by the RT-PCR method. Saliva samples showed similar or even higher positive rates than nasal swabs [22]. SARS-CoV-2 has been detected in stool (6.8–8.1 log_10_ copies per g of stool), and small concentrations were found in blood. On the other hand, urine samples do not show viral loads [23].

Saliva samples offer advantages as they can be collected directly by the patient, reducing the need and risk of exposure for healthcare professionals. Bergevin et al. found that when nasal and saliva samples were analyzed in patients with symptoms at 10 days by RT-PCR, the results were nearly identical (95%) [24]. Some articles have shown that ~90% of individuals infected with SARS-CoV-2 carry detectable viruses in their saliva [22,25,26]. In addition, a study carried out with 70 patients evaluated the correlation between nasopharyngeal swab specimens and saliva specimens concerning sensitivity by RT-PCR. The authors concluded that the saliva showed similar sensitivity and less variability compared to the nasopharyngeal sample [27].

## 3. Electrochemical Techniques Used in Disease Diagnostics

Electrochemical techniques, including potentiometry, amperometry, voltammetry, and electrochemical impedance spectroscopy, have been widely used for disease diagnostics [28]. Some advantages and disadvantages of the detection methods are mentioned in Table 1. A sensor can be classified both by the recognition element (chemically or biologically selective layer) and by the transducer used. Transducers are responsible for converting biological signals generated by physicochemical interactions on the surface of a sensor into a signal, usually electrical and measurable. Examples of transducers are electrochemical, piezoelectric, calorimetric, and optical. Therefore, an electrochemical device provides a response as a result of the interactions that occur on the detection surface, generating a signal variation in current, potential, conductivity, and impedance [29].

Taking into account a practical application, it is highly desirable to obtain biosensors and electrochemical sensors with high detection powers in nanograms, fentograms, or even lower concentrations [30,31,32,33,34]. To achieve such sensitivity, several factors should be taken into account so that an electrochemical approach achieves performance comparable to or even better than standard procedures. One of the crucial factors is the choice of electrode. As reactions are usually detected at the electrode surface, the electrode material and its dimensions must be taken into account, as well as its respective surface modifications, as these factors will directly influence its detection capability and the sensitivity of the method [35].

Currently, disposable screen-printed electrodes (SPEs) and electrochemical paper-based devices have stood out due to their low manufacturing cost, easy surface modification, and miniaturized dimensions that allow small sample volumes (a few microliters) [32,33].

Among the different approaches to electrochemical measurements, three are widely used. In the first approach, direct measurement of electroactive analytes can be used. The measurement of the signal generated by catalytically active analytes (enzymes), and indirect measurement using redox probes, such as ferri/ferrocyanide ([Fe(CN)_6_]^3−/4−^), ferrocene, and ruthenium, are other approaches [35]. The last one is widely used for the detection of viruses in electrochemical sensors. The main electrochemical techniques used for monitoring the detection of biomolecules in these devices are differential pulse voltammetry (DPV), square wave voltammetry (SWV), cyclic voltammetry (CV), or electrochemical impedance spectroscopy (EIS) [12,36].

## 4. Electrochemical Biosensors for Detection of SARS-CoV-2

Biosensors have been widely used for years as interesting tools for disease diagnostics and, more recently, for COVID-19 diagnostics. According to the International Union of Pure and Applied Chemistry (IUPAC), a biosensor is characterized by using a biological element capable of selectivity to recognize a target analyte. In contact with an electrochemical transducer, it must be able to recognize the biochemical reaction at its interface and convert it into a measurable electrochemical signal, which can be quantitative or semi-quantitative [29].

**Figure 2:**
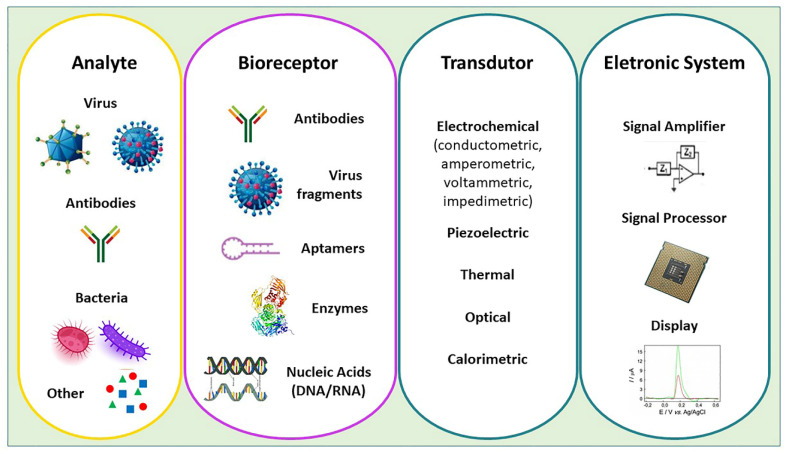
Components of an electrochemical biosensor.

The types of biomolecules that can be used are broad, and biosensors can be classified according to the type of interactions that occur with the bioreceptor: antibody/antigen, enzymes/ligands, nucleic acids/DNA [37]. Therefore, a biosensor consists of a biological recognition site, a transducer, and an electronic system that has a signal amplifier, a processor, and a display (Figure 2).

Generally, biosensors show pronounced selectivity, but their specificity is directly related to their construction. Therefore, some factors must be taken into account, such as the choice of the bioreceptor, the immobilization method, the choice of the most suitable enzymatic substrate, the transducer, as well as the electrochemical technique. These are the most important factors to consider when building an electrochemical platform [38].

### 4.1. Electrochemical Immunosensors

**Scheme 1:**
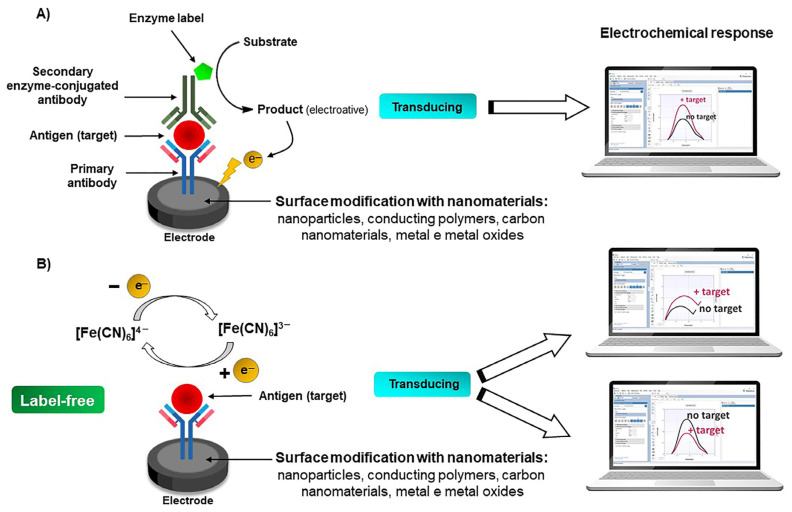
General representation of the main components of an electrochemical immunosensor. The scheme describes a generic immunosensor based on indirect (**A**) and direct (**B**) detection.

Immunosensors have been used for decades and represent a relevant percentage within the class of biosensors. The response of this device is related to the affinity between antibodies (Ab) and antigens (Ag). Thus, the recognition element immobilized on the electrode surface can be either an Ab or an Ag [39]. In this approach, it is most commonly used as a final step to inject an enzyme-labeled secondary antibody that follows the addition of a suitable enzyme substrate, as illustrated in Scheme 1A. The detection occurs indirectly through an enzymatic reaction that leads to the production of a molecule that is electroactive, providing an electrochemical signal [38].

The secondary enzyme-labeled antibody can be obtained commercially or obtained through conjugation protocols. Examples are horseradish peroxidase, alkaline phosphatase, and conjugated antibodies based on a mixture of H_2_O_2_ and 3,3′ and 5,5′-tertramethyl benzidine. A widely used enzyme substrate is 1-naphthyl phosphate, which is converted to electroactive 1-naphthol [40]. However, the choice of enzyme in the conjugated antibody must be considered, since it directly influences the technique to be used in the measurements, the pH of the medium, the electrode passivation effect, and the reaction time. Given these limitations, the development of marker-free and reagent-free electrochemical immunosensors has become increasingly common [38].

Electrochemical biosensors also allow for direct detection (label-free) through the physical phenomenon that occurs when the bioactive species recognizes the substrate (Scheme 1B). By direct determination, it is possible to measure any electrochemical property of the electrode, e.g., in an impedimetric biosensor, when the interaction between the analyte and the recognition site occurs, there is a partial blockage of the electroactive surface, resulting in an increase in the resistance to electron transfer, allowing the direct measurement of the capacitance or electrical resistance of the electrode [5,41,42].

**Figure 3:**
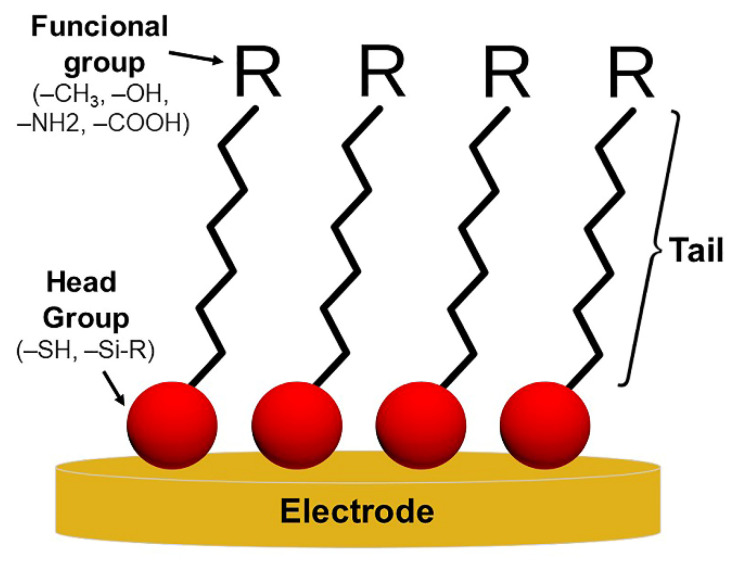
Generic illustration of self-assembled monolayers (SAMs).

The step of immobilizing the recognition element directly on the electrode surface is crucial. Self-assembled monolayers (SAMs) have been widely used for immobilizing a variety of biomolecules through spontaneous chemisorption on a surface that may contain groups such as thiols, amines, acids, disulfides, and silanes (Figure 3) [43]. In the case of gold screen-printed electrodes, the formation of SAMs through thiol-gold chemistry is often used, followed by the immobilization of the recognition element directly on this thiol monolayer. Some bridging molecules can be used as helpers in this step, such as glutaraldehyde [38,42,44,45]. Another approach includes the use of different polymeric matrices containing reactive amino and hydroxyl functional groups that are suitable for immobilizing biomolecules, as well as functional nanomaterials (nanoparticles, carbon nanomaterials, hybrid nanomaterials) [46]. As a last synthesis step, BSA is often used to avoid nonspecific interactions on the electrode surface, acting as a blocker [5,30,34,40,42,45].

Several electrochemical immunosensors have been developed as a detection proposal for SARS-CoV-2. Rahmati et al. manufactured an immunosensor for the detection of SARS-CoV-2-specific viral antibodies (IgG/IgM) through the functionalization of disposable screen-printed carbon electrodes (SPCE) [30]. First, a layer of nickel hydroxide nanoparticles was directly electrodeposited on the SPCE, followed by the immobilization of protein S, which was used as a specific bioreceptor. The redox probe used was [Fe(CN)_6_]^3−/4−^ and the electrochemical response was monitored by the DPV technique. The linear calibration curve ranged from 1 fg mL^−1^ to 1 µg mL^−1^, and the signals obtained were inversely proportional to antibody concentrations. With a duration of 20 min for each test, the biosensor presented a low limit of detection (LOD), 0.3 fg mL^−1^. Furthermore, the biodevice was tested on positive and negative clinical samples, showing agreement with the results obtained by the ELISA procedure.

Hryniewicz et al. investigated the influence of different morphologies of polypyrrole (PPy) synthesized on steel mesh electrodes for diagnosing the immune response against SARS-CoV-2. The authors synthesized globular (PSS) and nanotubular (NT) polypyrrole, functionalized with gold nanoparticles (AuNPs) [42]. The immobilization of the SARS-CoV-2 N-protein was performed using the methodology of self-assembled thiol monolayers. The immunosensor was applied for the detection of anti-SARS-CoV-2 N-protein monoclonal antibodies in PBS medium using the EIS technique in the measurements. The calibration curve for N-protein for PPy:PSS/AuNPs and PPy-NTs/AuNPs ranged from 10 to 60 ng mL^−1^ and 0.4 to 8 ng mL^−1^, respectively. The greater surface area of PPy-NTs promoted better detectability of the biosensor, presenting a LOD of 0.4 ng mL^−1^ against 2.5 ng mL^−1^ for the PPy:PSS film. Due to the greater sensitivity of PPy-NTs, it was applied to human serum samples, and the results were confirmed by qRT-PCR.

**Figure 4:**
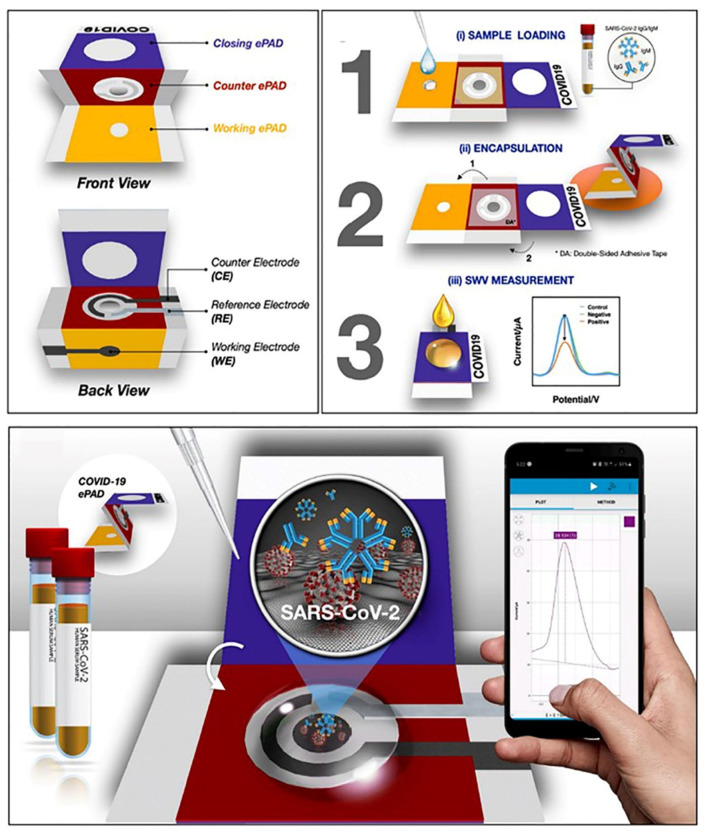
Electrochemical immunosensors for SARS-CoV-2 detection. Components and detection principle of the paper-based immunosensor (COVID-19 ePAD) for antibody detection. Reproduced with permission from [32]. Copyright 2021, Elsevier.

Yakoh et al. developed a paper-based electrochemical platform (COVID-19 ePAD) to detect antibodies (IgG/IgM) as well as an antigen (SARS-CoV-2 spike protein) (Figure 4) [32].

The ePAD COVID-19 test zone was constructed using graphene oxide. Thus, for antibody detection, RBD was immobilized to SARS-CoV-2 S-protein RBD, and for antigen detection, SARS-CoV-2 IgM antibodies were immobilized. The generated signal was inversely proportional to the target concentration and was monitored by the SWV technique using [Fe(CN)_6_]^3−/4−^ as a redox probe. A linear dynamic response for the detection of IgG/IgM, and S-protein was constructed in the range of 1–1000 ng mL^−1^. The LODs achieved by the proposed method were 0.11 ng mL^−1^ for S-protein, 0.96 ng mL^−1^ for SARS-CoV-2 IgG antibodies, and 0.14 ng mL^−1^ for SARS-CoV-2 antibodies IgM.

In another study, an immunosensor using a glassy carbon electrode (GCE) was developed [45]. First, Au-clusters were electrodeposited on GCE, and thiol sites were formed with cysteamine immobilization. Subsequently, glutaraldehyde was used as a bridge molecule for S-protein immobilization. Measurements were performed by SWV using [Fe(CN)_6_]^3−/4−^ as a probe, after an incubation time of 30 min. The proposed method achieved a LOD of 0.01 g mL^−1^, while the linear response ranged from 0.1 to 1000 ag mL^−1^. The sensor was applied to spiked samples of saliva and an oropharyngeal swab from healthy individuals, in which recovery values were obtained that ranged from 97% to 102%.

Mojsoska et al. describe an electrochemical immunoassay for the detection of S-protein [47]. The authors immobilized antispike antibodies on the surface of a screen-printed graphene electrode using 1-pyrenobutyric acid N-hydroxysuccinimide as a ligand. All electrochemical measurements were performed in PBS containing [Fe(CN)_6_]^3−/4−^. For S-protein detection, the SWV technique was used, requiring an incubation time of 45 min at 37 °C with 300 rpm agitation. The sensor was able to detect a specific signal above 260 nmol L^−1^ (20 µg mL^−1^) of S-protein and showed a linear range between 260 and 1040 nmol L^−1^, but LOD and LOQ values were not furnished in this study. 

Mehmandoust et al. developed a MOF-based immunosensor using the S-protein as a biomarker of COVID-19 [5]. The fabrication of the biosensor consisted of the modification of a screen-printed carbon electrode (SPCE) with a SiO_2_@UiO-66 core-shell nanocomposite. Subsequently, glutaraldehyde was deposited as a docking group for angiotensin-converting enzyme 2 (ACE2). Measurements were made by the EIS technique in the presence of [Fe(CN)_6_]^3−/4−^ as a redox probe, and a consistent response current was obtained after an incubation time of 5.0 min. The manufactured immunosensor showed a wide dynamic range, ranging from 100.0 fg mL^−1^ to 10.0 ng mL^−1^, with a LOD of 100.0 fg mL^−1^. Nasal samples were analyzed as a proof of concept, and the results were in agreement with the results (positive) obtained by RT-PCR, confirming the reliability of the developed immunosensor.

Another proposal for an impedimetric biosensor for the detection of SARS-CoV-2 S-protein was reported by Witt et al. [41]. In this work, the authors used thin films of boron-doped diamond (BDD). Anti-SARS-CoV-2 S1 antibodies were immobilized on the BDD surface previously modified with (3-aminopropyl)trimethoxysilane. Subsequently, the electrode was biotinylated, and then the streptavidin was immobilized. Finally, biotinylated anti-SARS-CoV-2 S1 antibodies were fixed on the BDD. EIS measurements were performed in a ferricyanide electrolyte solution. The linear range of the calibration curve for S-protein in PBS was 1–7 fg mL^−1^, and LODs close to 1 fg mL^−1^ were obtained. However, high relative standard deviations were obtained, and a low number of replicates were evaluated (n = 2). One should note that biosensors were not applied to the analysis of clinical samples, and only a culture medium used to store real human samples of SARS-CoV-2 as a complex media was analyzed. The detectability of the method was substantially decreased in this complex medium, as observed by higher LOD values ranging from 7.2 to 26.0 fg mL^−1^. Similar results have been observed by other authors, where the detectability was decreased in real samples when compared to the measurements in the PBS medium [9,40].

In some cases, the electrode is not used as an immobilization support. For example, sometimes the recognition element is immobilized on a separate support (magnetic- and nanobeads) and placed on the surface of an unmodified electrode as a final step, acting only as a measurement platform. In these cases, the electrode does not play a very crucial role in the detection, as it does not need to have properties to fix biomolecules [33,40].

**Figure 5:**
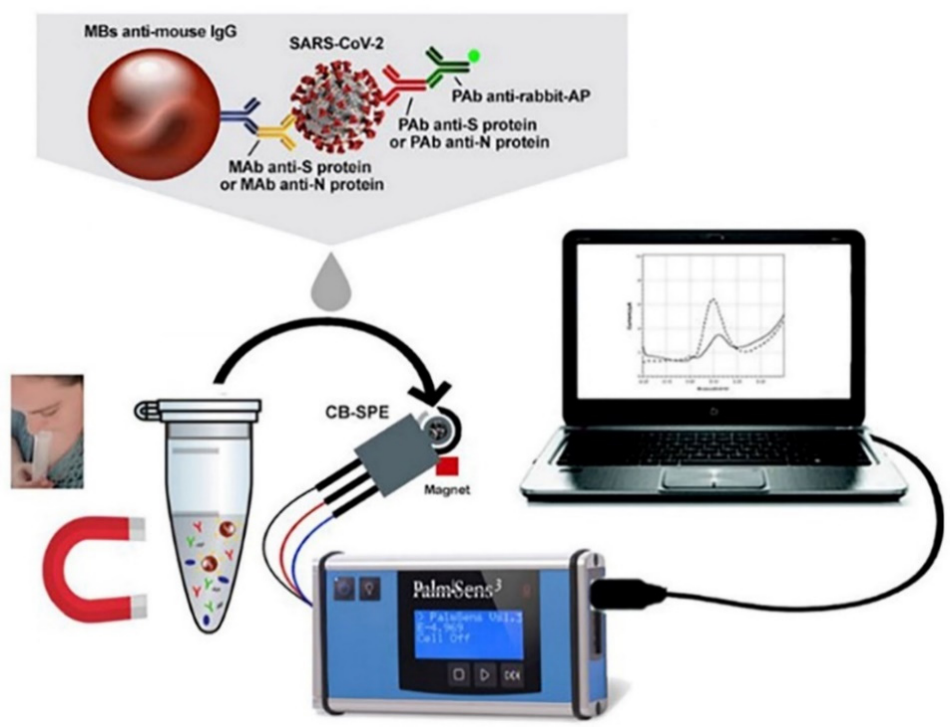
Electrochemical immunosensors for SARS-CoV-2 detection. Immunosensor based on SPE modified with magnetic beads (MBs) for antigen detection. Reproduced with permission from [40]. Copyright 2021, Elsevier.

Magnetic beads (MBs) have advantages in immunoassays, such as the possibility of loading a high amount of antibodies, allowing for an augmented sensitivity, preconcentration, and reducing the matrix effect in the measurement. Fabiani et al. developed an SPE-based electrochemical immunosensor modified with magnetic beads (MBs) for the detection of SARS-CoV-2 antigen (Figure 5) [40]. The proposed synthesis was used to obtain two immunoassay procedures, one for S-protein and the other for N-protein. The authors used the sandwich assay approach, immobilizing antibodies to S or N proteins in MBs. The coated and blocked MBs were placed in contact with the samples and subsequently in a solution containing secondary antibodies labeled with alkaline phosphate enzymes. After establishing the immunoassay, the beads containing the antigen were pre-concentrated on the surface of an SPCE (unmodified) with the aid of a magnet positioned below the working electrode. Then, 1-naphthyl phosphate was added as an enzymatic substrate, and the electroactive product formed (1-naphthol) was measured by applying the DPV technique. The analytical features of the electrochemical assay for protein S and protein N showed calibration curves with sigmoidal behavior from 0.04 to 10 μg mL^−1^ and 0.01 to 0.6 μg mL^−1^, respectively. The LODs for saliva samples for proteins S and N were 19 ng mL^−1^ and 8 ng mL^−1^, respectively. The proposed method was validated by RT-PCR, and specificity tests were also evaluated.

A magnetic electrochemical sensor with high sensitivity for detecting the SARS-CoV-2 S-protein was developed by Nascimento et al. [33]. The proposal was based on the modification of MBs and AuNPs with ACE2. The two materials were in contact with the sample for 1 h and formed an S-protein bioconjugate (MB-ACE2/Spike/ACE2-AuNP), which subsequently magnetically adhered to the surface of an SPCE (unmodified). S-protein detection was achieved by the redox properties of gold in AuNPs, monitoring the cathodic peak current obtained by the DPV technique. The linear range was 0.0009 to 360 fg mL^−1^, and the LOD obtained was 0.35 ag mL^−1^. The spike detection in saliva samples correlated well with the RT-PCR technique.

FET-based biosensors have shown great potential in the field of clinical diagnosis as well as for point-of-care testing. The interest in these devices is mainly due to the high sensitivity that can be achieved, the extremely short measurement time, and the possibility of working with very small amounts of samples [48].

A FET (field effect transistor) is an active device with three terminals: source, drain, and gate. The operating principle of FETs is based on the application of an electric field to control the current flow. Conductivity control can be achieved by varying the potential at the source and drain electrodes [35]. FET devices are great for weak-signal and/or high-impedance applications, proving to be a useful option in the electrochemical biosensor field. Graphene is a semiconductor material widely used in FET-based biosensors, in part due to its possibility of modification but mainly due to its properties that include sensitivity, low noise, high carrier mobility, and a large specific area [49].

Seo et al. developed a FET-based biosensor to detect SARS-CoV-2 using S-protein as a biomarker. The device was obtained by coating graphene sheets with an S-protein-specific antibody [50]. The immobilization of the antibody occurred through the N-hydroxysuccinimide ester of 1-pyrenebutyric acid. The biosensor was able to detect S-protein concentrations of 1 fg mL^−1^ in buffer solution (PBS) and 100 fg mL^−1^ in clinical samples.

**Figure 6:**
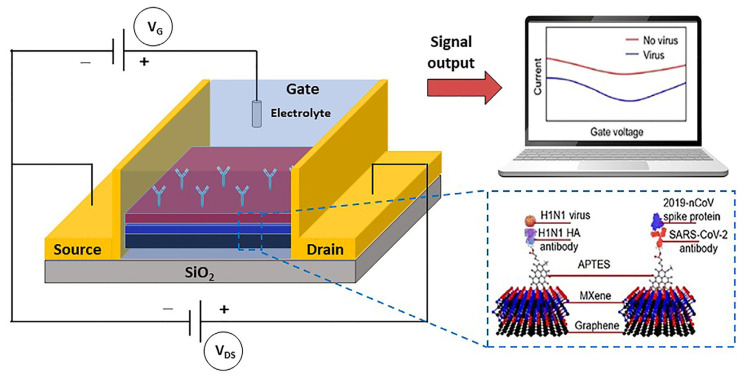
Circuit illustration of the Mxene–graphene FET sensor, antibody-antigen detection mechanism, and the signal obtained from a change in source current (adapted from [51]).

Another group of researchers also developed a FET sensor for the detection of SARS-CoV-2 [51]. The authors used graphene as a transduction material combined with Mxene (2-D transition-metal carbides). For the immobilization of the specific antibody of S-protein on the surface of Mxene-graphene, (3-aminopropyl)triethoxysilane was used as a linker (Figure 6). The calibration curve for the S-protein ranged from 1 fg mL^−1^ to 10 pg mL^−1^, and the LOD achieved was 1 fg mL^−1^ in PBS. 

Table 2 summarizes the electrochemical immunosensors for detecting SARS-CoV-2 mentioned in this review.

### 4.2. Electrochemical Genosensors and Aptasensors 

The use of nucleic acids as recognition elements has also been widely exploited as an important approach for obtaining high-affinity sensors. They offer some advantages, such as rapid production, low cost, long-term stability, resistance to high temperatures, ease of chemical modification after initial synthesis, and absence of host animals, thereby avoiding batch-to-batch variations [52]. Owing to these advantages, the interest in developing biosensors based on nucleic acids has increased significantly, which are commonly known as genosensors and aptasensors.

The classic nucleic acid biosensors, named genosensors, have an affinity principle the interaction of the single strand of DNA (ssDNA) with its complementary strand. It occurs by DNA hybridization, forming the well-known double helix (double helix DNA-dsDNA) that allows the detection of specific target genes. In this system, ssDNA acts as a probe [52,53].

**Scheme 2:**
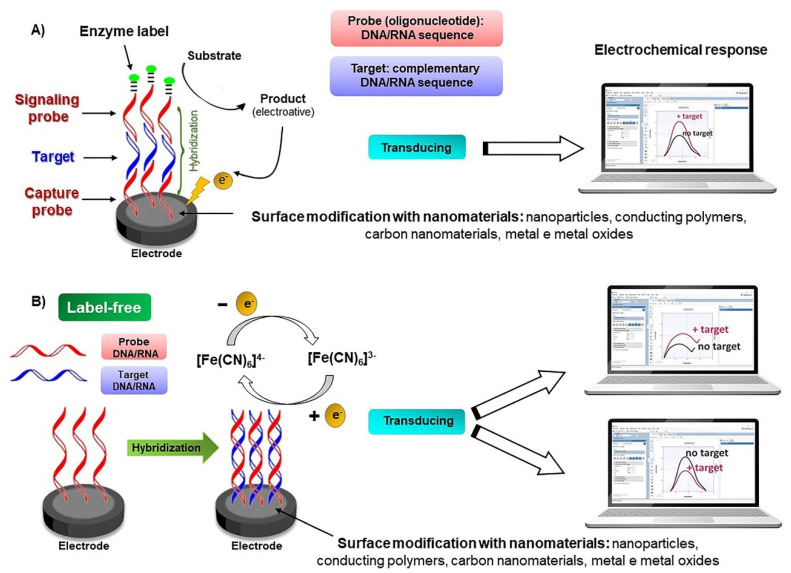
General representation of the main components and detection principles of classical (**A**) and label-free (**B**) electrochemical nucleic acid biosensors.

The analytical signal generated by a genosensor comes from the interaction between a probe (specific sequence), usually a small synthetic oligonucleotide, and a transducer (Scheme 2). The molecular probe works as a biorecognition element, and it is capable of detecting the complementary sequence of DNA or RNA. It is also important to mention that biorecognition occurs more efficiently when the degree of complementarity between the nitrogenous bases is greater between the two chains, which is generally obtained by the sandwich-type assembly. The better these interactions between nitrogenous bases of bioreceptors and target sequences are, the stronger and more specific the hybridization process will be [52,53,54].

The methods for immobilizing probes on the electrode surface depend on the type and nature of the transducer, which can be physical adsorption, chemical adsorption, affinity binding, or covalent binding. Among these, the simplest is physical adsorption, characterized by electrostatic interaction between negative charges on the probe and positive charges on the electrode surface. Chemical adsorption is based on the formation of nanostructured films (SAMs). A practical example of affinity binding (noncovalent) is the avidin or streptavidin-biotin complex, for which the probe is modified with biotin and the electrode is modified with avidin or streptavidin. In covalent bonding, the probe is modified at the end with reactive functional groups that have an affinity with the working electrode, reducing the steps for preparing the genosensor [53,54,55].

Electrochemical detection methods in genosensors can occur in two ways: (I) direct detection, which is the measurement of the electrical response of the target sequence, caused by the oxidation or reduction of nitrogenous bases during hybridization (Scheme 2A); or (II) indirect detection, in which electroactive compounds attached to the double helix are monitored (Scheme 2B) [52,55].

As an example of electrochemical genosensors for the diagnosis of COVID-19, it can be cited in the study developed by Li’s group [56]. The electrochemical biosensor developed is a super sandwich based on graphene functionalized with p-sulfolix [8] arene (SCX8) (SCX8-RGO) to enrich toluidine blue (TB) for the detection of SARS-CoV-2 RNA. AuNP and RGO were used in order to improve the sensitivity of the biosensor. DPV was used as an electroanalytical technique. After a period of 3 h of incubation, the TB electrochemical signal was detected in less than 10 s by a handheld smartphone. The linear analytical curve varied from 10 amol L^−1^ to 1 pmol L^−1^ and the LOD reached 3 amol L^−1^. Although the method had good sensitivity, the extensive time of contact with the sample was certainly the main disadvantage of the method.

**Figure 7:**
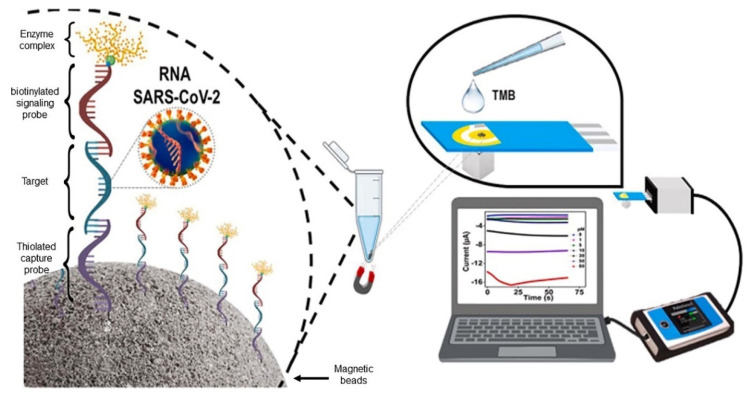
Nucleic acid-based electrochemical biosensors for SARS-CoV-2 detection. SPAuE-based sandwich-type genosensor modified with magnetic beads for RNA detection. Reproduced with permission from [57], with modifications. Copyright 2022, Elsevier.

Cajigas et al. developed an electrochemical genosensor using a screen-printed gold as a bare electrode (SPAuE) [57]. The assembly of the biosensor was performed on magnetic spheres functionalized with maleimide, which allowed the immobilization of the thiolated capture probe. In a sandwich-type system, hybridization occurs between the modified particles and the target probe (SARS-CoV-2 RNA) linked to a biotinylated signaling probe (Figure 7). To obtain an electrochemical response, an enzymatic complex based on streptavidin-horseradish peroxidase was connected to the signaling probe, completing the biosensor platform. The linear range obtained was 0–80 pmol L^−1^ and the LOD was 0.8 pmol L^−1^. The response was monitored by the chronoamperometry of the streptavidin-HRP enzymatic reaction after 60 s.

Song et al. reported the construction of a genosensor using electropolymerized polyaniline (PANI) nanowires [58]. Some advantages were presented by the authors in using the conductive polymer, such as the abundance of amino groups and the ease of changing parameters during electroplating, allowing a controlled formation of the desired structures. The authors obtained PANI nanowires electropolymerized in GCE, and the amino group’s biotin peptides were immobilized. For the immobilization of the target-specific biotin probe (SARS-CoV-2 RNA) to the biotin peptides, streptavidin was used as a linker. Thus, the biotin-streptavidin affinity system was used in the detection of the developed genosensors. Measurements were performed by DPV after 1 h of incubation, monitoring the change caused by hybridization. The biosensor demonstrated a linear range between 10 fmol L^−1^ and 1 nmol L^−1^, and a LOD of 3.5 fmol L^−1^. Table 3 summarizes the electrochemical genosensors for detecting SARS-CoV-2.

Biosensors that utilize aptamers as identification elements are called aptasensors. Aptamers consist of small oligonucleotides or peptides of the single chain (RNA or ssDNA—single stranded DNA) that bind to specific target molecules with high selectivity and specificity. One of the most commonly used methods for obtaining aptamers is SELEX (systematic evolution of ligands by exponential enrichment). They are created by contacting the target of interest with a set of random sequences of single-stranded oligonucleotides, but they can also be obtained naturally [59].

The use of aptamers as recognition molecules in the diagnosis of COVID-19 has great advantages. It can be cited the smaller size of aptamers, typically approximately 2–3 nm in diameter (30–60 nucleotides) compared to antibodies (12–15 nm in diameter) [60], which favors a lower steric hindrance on the surface of SARS-CoV-2 (65–125 nm in diameter) [61]. Such a feature allows the binding of more recognition elements on the SARS-CoV-2 surface, which leads to improved detection performances. Furthermore, aptamers have stability over a wide range of pH and temperature, in addition to having lower production costs than antibodies [59].

**Figure 8:**
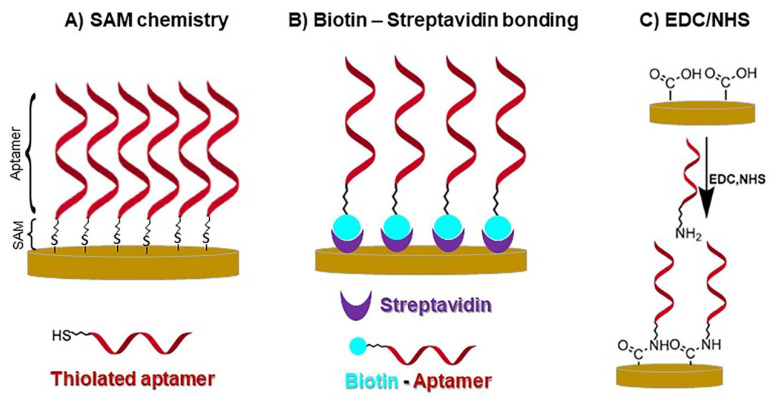
Schematics of the most common methods of surface immobilization for aptamers. (**A**) SAM chemistry, (**B**) biotin-streptavidin bond, and (**C**) EDC/NHS.

The immobilization of aptamers is a fundamental step, and it directly influences the sensitivity of the device. The main immobilization strategies can be described by physical (adsorption, occlusion, or encapsulation) and chemical methods (covalent bonding and crosslinking). It has been well reported that the greater the electroactive area for the deposition of aptamers, the better the sensitivity. For this reason, some nanomaterials are used to increase the active surface area of the electrode, such as AuNP, carbon nanotubes, graphene, polymeric films, and metallic oxides [52,62]. Figure 8 shows the most commonly used aptamer immobilization techniques [63].

Sari et al. reported an electrochemical aptasensor using an AuNP-modified SPCE to detect SARS-CoV-2-RBD [34]. The authors used the streptavidin-biotin system covalently bonded to the surface of SPCE modified with AuNP with the aid of the ligand 3-mercaptopropionic acid (MPA). The interaction between the aptamer and RBD-SARS-CoV-2 occurs through the formation of hydrogen bonds. The obtained responses were evaluated by DPV using a [Fe(CN)_6_]^3−/4−^ solution as a redox probe. The calibration curve ranged from 10 to 50 ng mL^−1^, and a LOD of 2.63 ng mL^−1^ was achieved. The electrochemical aptasensor showed optimal responses with an incubation time of the RBD-SARS-CoV-2 protein of 1 h. The biosensor was applied to saliva samples.

In another study, an impedimetric aptasensor was developed for the determination of SARS-CoV-2 RBD [64]. Initially, carbon nanofibers (CNFs) were decorated with AuNPs, and the formed nanocomposite was deposited on an SPCE (SPCE/CNF–AuNP). Finally, the thiol-terminal aptamer probe was anchored onto the SPCE/CNF-AuNP via an Au-S covalent bond. For SARS-CoV-2 RBD measurements, an incubation time of 40 min was required, and [Fe(CN)_6_]^3−^/^4−^ was used as a redox probe. The proposed method showed a linear range between 0.01 and 64 nmol L^−1^ (0.35–2240 ng mL^−1^) and a LOD of 7.0 pmol L^−1^ (0.24 ng mL^−1^). The analytical performance of the aptasensor was studied in spiked human saliva samples.

**Figure 9:**
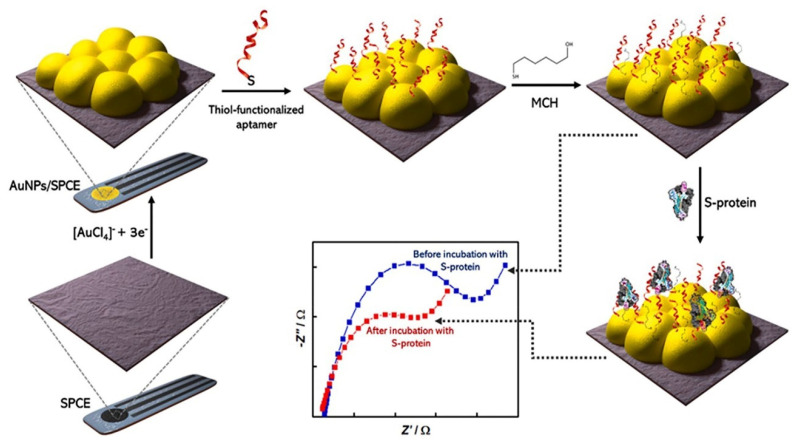
Nucleic acid-based electrochemical biosensors for SARS-CoV-2 detection. Fabrication steps of an aptasensor for SARS-CoV-2 S-protein detection. Reproduced with permission from [65]. Copyright 2022, Elsevier.

Abrego-Martinez et al. also developed an impedimetric aptasensor [65]. The immobilization of aptamer on gold nanoparticles deposited in SPCE was achieved throughSAM (Figure 9). SARS-CoV-2 S-protein detection was accomplished through impedimetric responses in a redox probe ([Fe(CN)_6_]^3−/4−^) in PBS (pH = 7.4) as a supporting electrolyte. The reading time was estimated after 40 min of incubation of the target in the probe. The calibration graph showed a linear range of 10 pmol L^−1^ to 25 nmol L^−1^ with a LOD for SARS-CoV-2 S-protein of 1.30 pmol L^−1^ (66 pg mL^−1^). The biosensor showed selectivity toward other coronaviruses. The sensor has not been applied to clinical samples.

Idili et al. used gold-wire electrodes for RBD detection [66]. The authors modified the surface of the electrodes, immobilizing specific aptamers, by the SAM method through interactions based on thiol. Atto MB2 (derived from methylene blue) was used as a redox reporter, generating an electrochemical signal that was measured by SWV. The authors reported that the sensor signal exhibits a strong dependence on the SWV frequency. The sensor response was recorded after 15 s of contact between the biosensor and the analyte, and a dynamic range was obtained between 760 pg mL^−1^ to 76 ng mL^−1^. The aptasensor showed specificity towards other coronaviruses and other proteins, and samples of serum and artificial saliva were used to confirm the potential of the sensor for the clinical diagnosis of COVID-19.

**Figure 10:**
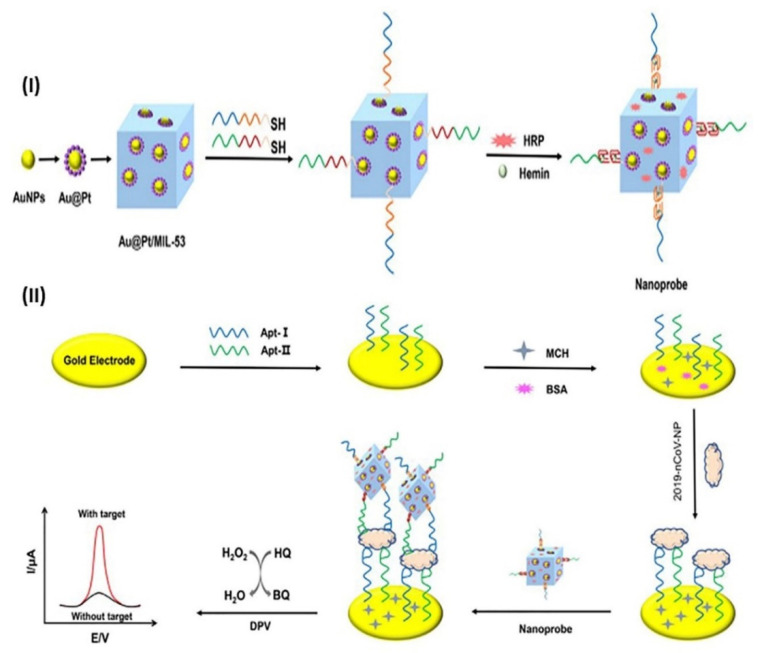
Nucleic acid-based electrochemical biosensors for SARS-CoV-2 detection. Fabrication process of the nanoprobes (**I**) and the aptasensor for capture and detection of SARS-CoV-2 nucleocapsid protein (**II**). Reproduced with permission from [67]. Copyright 2021, Elsevier.

Tian et al. synthesized metal-organic structures (MOFs) decorated with nanoparticles (Au@Pt) and enzymes to determine the N-protein (which acted as a nanoprobe), and immobilized double aptamers on the surface of a gold electrode through SAM (Figure 10) [67].

The main aim of the nanoprobe was to amplify the generated signal, improving sensitivity. Therefore, N-protein detection was based on a sandwich assay (aptamer-protein-nanoprobes). Quantification was performed by DPV, and measurements were performed after 2 h of incubation. A linear range of 0.025–50 ng mL^−1^ and a LOD of 8.33 pg mL^−1^ were obtained for N-protein using the aptasensor. The human serum samples were analyzed, and the results obtained by the developed aptasensor were concordant with the ELISA results.

Table 4 summarizes the electrochemical aptasensors for detecting SARS-CoV-2.

## 5. Electrochemical Sensors for the Detection of SARS-CoV-2

### MIP-Based Electrochemical

Molecularly imprinted polymers (MIPs) belong to the class of biomimetic materials, and their application in the field of bioanalysis is growing quickly. In the literature, the versatility of MIPs for imprinting small and large molecules, including proteins [9,31] and intact viruses [6,68], has been well established. The interest in these materials is attributed mainly to their affinity and ability to selectively recognize a particular target molecule (template), which resembles natural receptors. The high selective affinity of MIPs is intrinsically attributed to their three-dimensional structure and chemical composition [69].

**Scheme 3:**
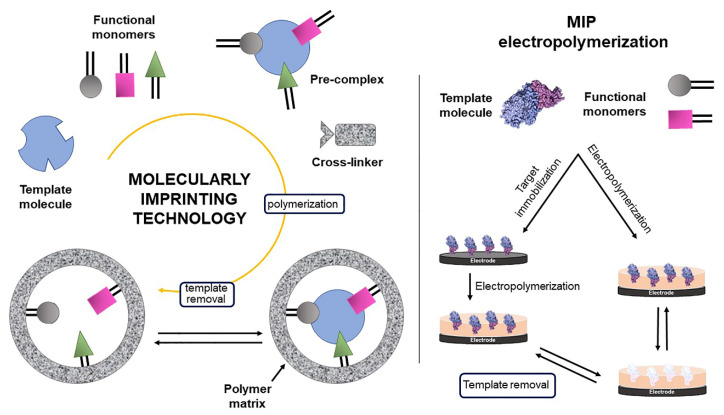
Schematic diagram of the molecular imprinting process.

Overall, the specific recognition characteristics of MIPs are attributed to the cavities formed in the polymer, which are complementary in size and shape to the analyte of interest. This interaction resembles affinity-based biological recognition systems, such as the antigen-antibody and enzyme-substrate systems. Briefly, MIPs are prepared by copolymerization of functional monomers in the presence of a template and crosslinkers. When MIP is obtained by electropolymerization, crosslinkers are not necessary (Scheme 3). Template removal can be performed with solvents, acids, bases, or even by applying an electrochemical potential to these media. Removing the template from the polymeric matrix leads to the formation of cavities called binding sites that have a complementary size and shape to the template, as well as functional groups with an affinity for the target analyte, thus generating a molecular memory. In addition to being selective, these materials are stable in strongly acidic and basic media, in organic solvents, and at high temperatures [69].

Integrating molecular imprinting technology with electrochemical techniques is a valuable approach to disease diagnosis. Usually, the principle of measuring MIP-based electrochemical nanosensors is based on the changes in current or charge resistance transfer in the presence and absence of the target analyte using a redox probe at the thin film of MIP deposited on the surface of a bare electrode.

The electropolymerization mechanism has been widely used to obtain thin polymeric films on electrode surfaces as it allows controlled and rapid deposition with adjustable thickness. Active redox functional monomers such as phenols, dopamine, o-phenylenediamine, acrylamide, pyrrole, and aniline are used in this approach [6,70,71].

**Figure 11:**
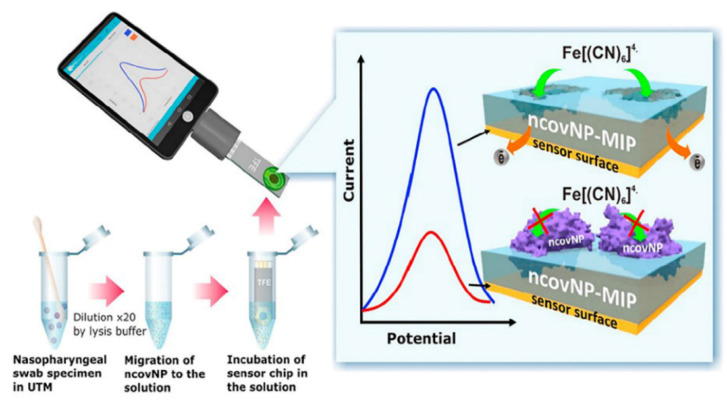
MIP-sensor for COVID-19 diagnosis (ncovNP detection). Principle of diagnosis and detection. Reproduced with permission from [31]. Copyright 2021, Elsevier.

Usually, CV is used to induce functional monomer polymerization on the electrode surface, and polymer film growth is directly related to the number of sequential cycles used. When the template is removed, selective binding sites are obtained to rebind the target. The rebinding is commonly monitored by CV, DPV, EIS, or SWV. As mentioned, usually a small inorganic redox marker is used to probe the effect of the presence of the target template on the polymer surface. In the absence of the target analyte, the redox probe furnishes a measurable and characteristic current signal. It occurs due to unoccupied cavities in the MIP, which makes the MIP surface more susceptible to electron transfer to the redox probe. On the other hand, in the presence of an analyte, the cavities are filled, thus decreasing the charge transfer of the redox probe, and as a consequence, leads to a reduction in the current signal (Figure 11). When the concentration used in rebinding is significantly high, a saturation of the interaction sites can occur, leading to surface passivation and unresponsiveness, where no current is observed [31,70,71,72].

MIP-based sensors have been used in the diagnosis of COVID-19. For instance, a MIP-based sensor for the detection of SARS-CoV-2 RBD was developed by Tabrizi et al. using macroporous gold screen-printed electrodes [73]. MIP was electropolymerized on the electrode surface using o-phenylenediamine (functional monomer) in the presence of SARS-CoV-2-RBD molecules (template). Template removal was carried out using alkaline ethanol. Each synthesis step was characterized by a CV. The EIS technique was used to perform measurements and [Fe(CN)_6_]^3−/4−^ was used as a redox probe. The sensor showed a linear response from 2.0 pg mL^−1^ to 40 pg mL^−1^ with a LOD value of 0.7 pg mL^−1^ (20 fmol L^−1^). The MIP sensor was applied to saliva samples, and the results did not show a significant difference when compared to the ELISA method.

Raziq et al. reported the first biomimetic sensor for the detection of the SARS-CoV-2 N-protein (Figure 10) [31]. The MIP was prepared after the covalent immobilization of N-protein on a 4-aminothiophenol (4-ATP)-modified gold electrode. The functional monomer m-phenylenediamine was defined by computational modeling. Thus, the poly-m-phenylenediamine film was electrodeposited. The polymeric film cavities were formed after treatment with an ethanolic solution and acetic acid. All the steps of the MIP preparation were characterized by the redox probe ([Fe(CN)_6_]^3−/4−^). DPV was used as a measurement technique. The calibration plot of the MIP sensor showed a linear range for low concentrations of N-protein (2 to 111 fmol L^−1^) and a LOD of 15 fmol L^−1^. The authors also showed, through selectivity studies, the ability of the sensor to differentiate the relationships of several proteins. The sensor was validated by the RT-PCR technique, which showed a positive correlation with the responses obtained from nasopharyngeal swab specimens.

Another research group also developed a sensor using the N-protein as a template [74]. A SPCE was modified with gold/graphene (Au/Gr) nanohybrids and used arginine as a functional monomer to immobilize N-protein. To study the feasibility of applying the sensor in the diagnosis of SARS-CoV-2, real and artificial samples were used. The evaluated samples were enriched and showed recovery percentages ranging from 94.3% to 102.0%. The working range showed linearity from 10.0–200.0 fmol L^−1^, with a low LOD of 3 fmol L^−1^.

Ayankojo et al. reported the synthesis of a MIP-based electrochemical sensor for the diagnosis of COVID-19 using gold-based thin-film metal electrodes (Au-TFME) [9]. For MIP synthesis, the Au-TFME surface imprinting strategy was adopted. First, the electrode was modified with a ligand monolayer for S-protein immobilization. Then, a thin film of poly(3-aminophenylboronic acid) (APBA) was electrodeposited. Due to the ability of glycogen –B(OH)_2_ to form reversible covalent bonds, the use of APBA is suitable for protein recognition [70]. Template removal was carried out using dithiothreitol, followed by washing with acetic acid. Analyte measurement was possible in 15 min using SWV with the aid of the [Fe(CN)_6_]^3−/4−^ redox probe. The sensor was able to detect ncovS1 in nasopharyngeal swab samples with a LOD of 64 fmol L^−1^. The results obtained were validated by the RT-PCR method, and the sensor showed high selectivity in relation to other proteins.

A polypyrrole-based sensor (MIP-Ppy) for S-protein detection was proposed by Ratautaite et al. [71]. In a three-electrode system, the authors used a Pt disk as a working electrode platform for electrodepositing the polypyrrole film. Subsequently, the electrode was placed in a sulfuric acid solution to extract the template. Due to the ability of glycogen–B(OH)_2_ to form reversible covalent bonds, the use of APBA is suitable for protein recognition [70]. Template removal and the washing step were carried out using dithiothreitol and acetic acid, respectively. Chronoamperometry, in pulsed amperometric mode, was used as an electrochemical technique for target analyte detection. The calibration curve showed a linear range between 5 μg mL^−1^ and 25 μg mL^−1^. Selectivity tests were performed only with BSA. However, the limit of the detection of the method was not furnished, as well as the concentrations in the linear range were much higher than those of other studies reported in the literature. Furthermore, the authors did not apply the MIP-Ppy to clinical samples and did not validate the sensor with any reference clinical analysis technique.

The use of MIP-based sensors for the detection of whole viruses, known as virus-imprinted polymers (VIPS), instead of S and N proteins, has also been reported. However, it has been a challenge for the production of MIP-based whole virus detection strategies, which may be related to the great difficulty in producing selective cavities using whole viruses as a template, as a result of fragile architecture, large dimensions, and low stability in organic solvents [75].

The imprinting process requires a large number of pure viruses, appropriate laboratory, equipment, and experienced personnel for sample isolation and purification. Another factor that hinders the handling of viruses is the need for laboratories with certain levels of biosafety clearance [76]. Furthermore, the formation of heterogeneous binding sites and a greater likelihood of cross-reactions may occur due to the significant number of interaction sites and functional groups on the viral surface [77]. In this way, the proper choice of a synthesis route is fundamental and a critical step for the successful development of VIPs. There are different polymerization approaches for producing molecularly imprinted materials, such as mass imprinting (3-D) and surface imprinting (2-D) [78].

Reddy’s group developed an electrochemical sensor for the selective recognition of the whole SARS-CoV-2 virus [6]. Electrochemical deposition of MIP took place on the surface of SPCE using a solution containing N-hydroxymethylacrylamide as a functional monomer, N,N′-methylenebisacrylamide as a crosslinker, and SARS-CoV-2 pseudoparticles as a template. The virus was electrochemically extracted using a PBS solution. The EIS technique was used to evaluate the electrochemical responses and the redox couple [Fe(CN)_6_]^3−/4−^ was used as a probe. The MIP-sensor showed a response time of <10 min with a linear dynamic range that was found to be log_10_ 3.0–7.0 pfu mL^−1^ and a LOD of 4.9 log_10_ pfu mL^−1^. Furthermore, 24 real saliva samples from patients were analyzed. Overall, positive and negative cases had a 75% agreement between the MIP-sensor method and the LAMP method.

Hussein et al. also developed a MIP-based impedimetric sensor. The authors imprinted the SARS-CoV-2 viral particles on screen-printed electrodes CNTs/WO_3_ [72]. The polymeric film of poly(meta-aminophenol) was obtained by the electrochemical polymerization method. EIS measurements were conducted using the redox couple [Fe(CN)_6_]^3−/4−^. The calibration curve was 7.0–320.0 pg mL^−1^, although the LOD was 57.0 pg mL^−1^. The selectivity study showed that the developed sensor was capable of discriminating SARS-CoV-2 from different respiratory syndrome viruses, with a low interference response (>40%). Thus, the sensor was successfully applied to 23 samples of nasopharyngeal swabs, considering RT-PCR as a reference method. Diagnosis by the proposed method can be accurately provided in 5 min.

Other researchers have used a different approach [68]. The authors synthesized and characterized a material with a viral imprint of SARS-CoV-2, and subsequently, a suspension of the material was deposited on a GCE. For this purpose, graphene oxide flakes were synthesized and decorated with an interconnected polypyrrole-boronic complex that has the potential to promote greater selectivity and specificity. Boronic acid forms covalent bonds with glycoproteins through the cis-diodes present in these molecules. Thus, the interaction with the analyte in the formed cavities occurs both by shape and by functional groups within the polymeric structure. Polymerization occurred in the presence of 3-APBA (crosslinker) and pyrrole (functional monomer). Template removal was performed using acetic acid, acetone, and ethanol.

The sensor preparation was based on a drop-casting method using aqueous suspensions of MIP and NIP. Each step-by-step sensor fabrication process was monitored by EIS and CV using a ferricyanide redox probe. To evaluate the sensor response for detecting SARS-CoV-2, DPV and amperometry techniques were applied using the same probe. Using the DPV technique, the response time for each measurement was 1 min, with a linear range from 0.74 to 9.03 fg mL^−1^ and a LOD of 0.326 fg mL. In the amperometric assay, the response was obtained in a shorter time (20 s), but a slight reduction in sensitivity was observed, and a linear range between 13.14 and 118.9 fg mL^−1^ and a LOD of 11.32 fg mL^−1^ were reported. The authors showed how the choice of electroanalytical technique can also influence the sensitivity of the response obtained. Table 5 presents the MIP-based sensors discussed in this review.

## 6. Closing Remarks

Analysis of the reviewed studies indicates that the electrodes most commonly used for the diagnosis of COVID-19 are the SPEs and the GCE. To obtain more portable, miniaturized, and simpler devices, SPEs are a viable alternative, as they are configured in such a way as to compose the three electrodes (work, reference, and auxiliary) on a single substrate. Furthermore, SPEs allow for analysis via portable potentiostats using smartphones.

When considering the various options for SPEs, it is observed that SPCEs are widely used, as reported in the literature. The fact that it is possible to modify its surface is pointed out as a justification for this preference. One of the main modifications carried out is the incorporation of AuNP, which allows the anchoring of biomolecules by means of different immobilization techniques, especially SAMs. These modifications can be seen as a way to reduce sensor costs, since commercially purchased SPAuE electrodes have a high cost, while modifying SPCEs or GCEs with AuNP is a more economical alternative.

It is possible to infer that most of the platforms presented in this review offer more straightforward approaches due to the absence of a target modification step and the label-free nature of their signaling mechanisms (i.e., using an active redox probe [Fe(CN)_6_]^3−/4−^).

The electrochemical sensors/biosensors can offer high detection power (LODs < 10 fg mL^−1^) and selectivity similar to the gold standard technique (RT-PCR), as well as fast analysis (<5 min) (Table 1, Table 2 and Table 4). Some studies reported the LOD values in PBS (a medium generally used for the development of the method) and in real samples. It was observed in higher LOD values in clinical samples or media that simulate a more complex environment, thereby clearly demonstrating a matrix effect, although the selectivity is assured. These studies highlight the importance of evaluating sensor performance not only in standard media but also in real samples. Performing the analysis on real samples is considered crucial to validating the efficiency of the proposed method, as evidenced in the literature.

In these methods, mainly S or N proteins and IgG and IgM antibodies have been used to develop sensors and biosensors, while saliva samples were mostly used in clinical samples in the reported studies. This is because saliva is an aqueous sample, which does not require prior preparation, usually requiring only dilution or even no procedure at all, compared to samples obtained through oropharyngeal or nasopharyngeal swabs.

It is possible to notice that the choice of the electrochemical technique directly influences the sensitivity, as seen in the work by Hashemi et al. [66]. In fact, for a similar probe applied to different techniques, the LODs can change by up to three orders of magnitude [39]. This statement shows that the choice of detection technique plays an important role in obtaining highly sensitive methods.

As observed in this review, among electrochemical techniques, voltammetric ones are the most used in the detection of biomolecules, especially DPV. The greater sensitivity due to the ability to differentiate the capacitive current justifies the preference for differential pulse and square wave techniques [28]. These techniques also offer a shorter measurement time compared to the EIS technique, which is also widely used.

Another essential point that must be taken into account is the manufacturing time of each sensor. Generally, immunosensors have longer steps, requiring hours to immobilize biomolecules. The stage of blocking nonspecific bindings with blocking solutions, such as BSA, takes a considerable amount of time—approximately 30 min. On the other hand, MIP-based sensors may have a reduced synthesis time, especially those using the electropolymerization approach. Template removal, when performed by immersing the electrode in extracting solutions, may represent one of the most time-consuming steps (a few hours). However, when the extraction is performed electrochemically, the formation of printed cavities can be achieved in a few minutes.

Therefore, in view of the above, electrochemical devices can be used as a tool for the rapid, selective, and sensitive diagnosis of SARS-CoV-2 in hospitals, airports, schools, and hotspot regions.

## 7. Conclusions

The aim of this paper was to provide a brief overview of biosensors or electrochemical sensors as sensing platforms for the diagnostics of COVID-19. The studies herein reported will be helpful to researchers who wish to develop biosensors or electrochemical sensors, guiding some paths through the explanation of the operation of each device herein presented. The positive and negative points of the different approaches are highlighted, as well as their inherent challenges.

It is essential to point out that for the development of these devices, it is necessary to have knowledge of molecular biology, and electrochemical characterization techniques, as well as seek to understand the detection mechanisms. In turn, after preparing these sensors and biosensors, there is an inherent advantage detected with the commonly used RT-PCR molecular detection methods.

Owing to the high cost, time-consuming process, and the need for highly qualified personnel to perform the RT-PCR tests, electrochemical sensors/biosensors for antigen detection, such as SARS-CoV-2, have been successfully developed and are considered viable as tools for the diagnosis and screening of COVID-19.

## Figures and Tables

**Table 1:** Characteristics of electrochemical techniques commonly used in antigen detection.

Electrochemical Techniques		Advantages	Disadvantages
Voltammetric (time-dependent potential resulting in current as a function of applied potential)	DPV	High sensitivity; high peak-shaped resolution; minimization of background effects and double layers.	Slower than SWV.
	SWV	Is slightly more sensitive than DPV; Is faster than DPV; Similar to DPV, it is able to discriminate the nonfaradaic currents; in the reverse cathodic process, the oxygen interference is avoided.	More evidence indicates a reversible redox process.
Amperometric (fixed potential resulting in an initial transient current)	Chrono-amperometry	High sensitivity; minimized signal-to-noise; easy coupling of flow systems.	Selectivity is low when using high positive and negative potentials
Impedimetric (varied frequency, response obtained in impedance x imaginary impedance)	(EIS)	Sensitive; direct detection of biomolecular recognition.	Longer measurement time compared to other techniques.
Potentiometric (potential that develops in an electrochemical cell containing two electrodes, an indicator, and a reference. A high-impedance potential measuring device is necessary.		Low cost; easy coupling of flow systems; wide linear range and good sensitivity.	Less precise; very sensitive to changes in the ionic strength; longer time to stabilize the measurements

DPV = differential pulse voltammetry; SWV = square wave voltammetry; EIS = electrochemical impedance spectroscopy.

**Table 2:** Summary of most methods developed for the detection of SARS-CoV-2 based on electrochemical immunosensors.

Biomarker	Electrode	Modifiers	Technique	Linear Range	Limit of Detection	Analysis Time	Ref.
Antibodies IgG/IgM	SPCE	Ni(OH)_2_NPs/S-protein	DPV	1 fg mL^−1^ to 1 µg mL^−1^	0.3 fg mL^−1^	20 min	[30]
Antibody anti-N protein	steel mesh	Ppy:PSS/AuNPs/3-MPA/EDC:NHS/N-protein	EIS	Ppy:PSS/AuNPs 10–60 ng mL^−1^	Ppy:PSS/AuNPs 0.386 ng mL^−1^	30 min	[42]
Antibody anti-N protein	steel mesh	Ppy-NTs/AuNPs/MPA/EDC:NHS/N-protein	EIS	Ppy-NTs/AuNPs 0.4–8 ng mL^−1^	Ppy-NTs/AuNPs 2.456 ng mL^−1^	30 min	[42]
Antibody anti-S protein	GCE	Au/Cysteamine/glutaraldehyde/S-protein	SWV	0.1–1000 ag mL^−1^	0.01 ag mL^−1^ in sample	30 min	[45]
Antibodies IgG/IgM	GO-ePAD	S-Protein RBD via EDC:NHS	SWV	1–1.000 ng mL^−1^	IgG—0.96 ng mL^−1^ IgM—0.14 ng mL^−1^	30 min	[32]
Protein S	GO-ePAD	IgM antibody via EDC/NHS	SWV	1–1.000 ng mL^−1^	0.11 ng mL^−1^	45 min	[32]
Protein S	SPCE	MOFs/Cysteamine/glutaraldehyde/ACE2	EIS	100 fg mL^−1^ to 10 ng mL^−1^	100 fg mL^−1^	5 min	[5]
Protein S	SPCE	MBs-ACE2/ S-protein/ACE2-AuNPs	DPV	0.0009–360 fg mL^−1^	0.35 ag mL^−1^	1 h	[33]
Protein S	BDD	APTMS/ biotin-straptavidin/anti-S antibody	EIS	1–7 fg mL^−1^	0.9–1 fg mL^−1^	5 min	[41]
Protein S	Graphene-FET	Graphene/Mxene/ APTES/anti-S antibody	potentiometry	1 fg mL^−1^ to 10 pg mL^−1^	1 fg mL^−1^ (PBS)	-	[51]
Protein S	SPGE	PBASE/anti-S antibody	SWV	260–1040 nmol L^−1^	-	45 min	[47]
Protein S	Graphene-FET	Graphene/PBASE/anti-S antibody	potentiometry	1 fg to 10 pg mL^−1^ (PBS) 100 fg to 100 pg mL^−1^ (sample)	1 fg mL^−1^ (PBS) 100 fg mL^−1^ (sample)	-	[50]
Protein S	SPCE	MBs/Mab anti-S protein/SARS-CoV-2/Pab anti-S protein/Pab anti-rabbit-AP	DPV	0.04–10 μg mL^−1^	14 ng mL^−1^ (PBS); 19 ng mL^−1^ (sample)	32 min	[40]
Protein N	SPCE	MBs/Mab anti-N protein/SARS-CoV-2/Pab anti-N protein/Pab anti-rabbit-AP	DPV	0.01–0.6 μg mL^−1^	4 ng mL^−1^ (PBS); 8 ng mL^−1^ (sample)	32 min	[40]

DPV: differential pulse voltammetry; EIS: electrochemical impedance spectroscopy; SWV: square wave voltammetry; FET: field-effect transistor; SPCE: screen-printed carbon electrode; NPs: nanoparticles; Ppy: polypyrrole; Ppy:PSS: globular polypyrrole; Ppy:NTs: nanotubular polypyrrole; GCE: glassy carbon electrode; ePAD: electrochemical paper-based analytical device; SPGE: screen-printed graphene electrode; BDD: boron-doped diamond; MOFs: SiO_2_@UiO-66; GO: graphene oxide; MBs: magnetic beads; PBASE: 1-pyrenobutyric acid N-hydroxysuccinimide; ACE2: angiotensin-converting enzyme 2; AP: alkaline phosphate; SAM: self-assembled monolayer; Mab: monoclonal antibody; Pab: polyclonal antibody.

**Table 3:** Summary of electrochemical genosensors for SARS-CoV-2 detection.

Biomarker	Electrode	Modifiers	Technique	Linear Range	Limit of Detection	Analysis Time	Ref.
RNA	SPCE	Au@Fe_3_O_4_/CP/RNA/ Au@SCX8-RGO-TB	DPV	10 amol L^−1^ to 1 pmol L^−1^	3 amol L^−1^	3 h incubation	[56]
RNA	SPAuE	MBs/maleimide/thiolated capture probe/RNA/ biotinylated signaling probe/streptavidin-horseradish peroxidase	Chron.	0–80 pmol L^−1^	0.8 pmol L^−1^	1 min	[57]
RNA	GCE	PANI/biotin-peptide/SA/biotin-probe	DPV	10 fmol L^−1^ to 1 nmol L^−1^	3.5 fmol L^−1^	1 h incubation	[58]

DPV: differential pulse voltammetry; Chron.: chronoamperometry; SPCE: screen-printed carbon electrode; SPAuE: screen-printed gold as bare electrode; GCE: glassy carbon electrode; MB: magnetic beads; SA: streptavidin; PANI: polyaniline nanowires.

**Table 4:** Summary of electrochemical aptasensors for SARS-CoV-2 detection.

Biomarker	Electrode	Modifiers	Technique	Linear Range	Limit of Detection	Analysis Time	Ref.
RBD	SPCE	AuNPs/MPA/EDC:NHS/ streptavidin/biotinylated aptamer	DPV	10.0 to 50.0 ng mL^−1^	2.63 ng mL^−1^	1 h	[34]
RBD	SPCE	CNF@AuNPs/thiolated aptamer	EIS	0.01 to 64 nmol L^−1^ (0.35 to 2240 ng mL^−1^)	7.0 pmol L^−1^ (0.24 ng mL^−1^)	40 min	[64]
RBD	SPCE	AuNPs/thiolated aptamer	EIS	10.0 pmol L^−1^ to 25.0 nmol L^−1^	1.30 pmol L^−1^ (66 pg mL^−1^).	40 min	[65]
RBD	Gold wire electrode	Thiolated aptamer-Atto MB2	SWV	760 pg mL^−1^ to 76 ng mL^−1^	-	5 min	[66]
protein N	GE	Thiolated aptamer/N-protein/thiolated aptamer/Au@Pt/MOF	DPV	0.025 to 50 ng mL^−1^	8.33 pg mL^−1^	2 h	[67]

DPV: differential pulse voltammetry; EIS: electrochemical impedance spectroscopy; SWV: square wave voltammetry; SPCE: screen-printed carbon electrode; GE: gold electrode; CNF: carbon nanofibers; MOF: MIL-53.

**Table 5:** Summary of MIP-based biomimetic sensors for SARS-CoV-2 detection.

Transducer	Template/Monomer	Technique	Linear Range	Limit of Detection	Analysis Time	Ref.
SPCE	SARS-CoV-2 pps/NHMA; MBAm (crosslinker)	EIS	3.0 to 7.0 log_10_ pfu mL^−1^	4.9 log_10_ pfu mL^−1^	<10 min	[6]
CNTs/WO3-SPCE	SARSCoV-2 whole virus/m-AP	EIS	7.0 to 320 pg mL^−1^	57 pg mL^−1^	5 min	[72]
GCE	SARS-CoV-2 whole virus/(GO) Pyrrole; PBA (crosslinker)	DPV	0.74 to 9.03 fg mL^−1^	0.33 fg mL^−1^	1 min	[68]
GCE	SARS-CoV-2 whole virus/(GO) Pyrrole; APBA (crosslinker)	Amp.	13.14 to 118.9 fg mL^−1^	1.32 fg mL^−1^	20 s	[68]
MP-Au-SPE	SARS-CoV-2 RBD/ o-PD	DPV	2 to 40 pg mL^−1^	0.7 pg mL^−1^ (20 fmol L^−1^)	20 min	[73]
Pt electrode	S-protein/Pyrrole	Chron. in pulsed Amp. (PAD)	5 μg mL^−1^ to 25 μg mL^−1^	-	-	[71]
4-ATP/Au-TFME	S-protein/APBA	SWV	PBS—26.7 to 194 fmol L^−1^ Sample—0 to 400 fmol L^−1^	15 fmol L^−1^ (PBS) 64 fmol L^−1^ (sample)	15 min	[9]
4-ATP/Au-TFE	N-protein/m-PD	DPV	2 to 111 f mol L^−1^	15 fmol L^−1^	-	[31]
Au/Gr/SPCE	N-protein/Arginine	DPV	10 to 200 f mol L^−1^	3.0 fmol L^−1^	-	[74]

Chron.: chronoamperometry; Amp.: amperometry; DPV: differential pulse voltammetry; EIS: electrochemical impedance spectroscopy; SWV: square wave voltammetry; MP-Au-SPE: macroporous gold screen-printed electrode; Au-TFME: gold-based thin-film metal electrode; o-PD: orthophenylenediamine; m-PD: metaphenylenediamine; APBA: 3-aminophenylboronic acid; NHMA: N-hydroxymethylacrylamide; MBAm: N,N′-methylenebisacrylamide; GO: graphene oxide; SARS-CoV-2 pps: SARS-CoV-2 pseudoparticles; m-AP: meta-aminophenol.

## Data Availability

Not applicable.
